# The Disturbed Iron Phenotype of Tumor Cells and Macrophages in Renal Cell Carcinoma Influences Tumor Growth

**DOI:** 10.3390/cancers12030530

**Published:** 2020-02-25

**Authors:** Matthias Schnetz, Julia K. Meier, Claudia Rehwald, Christina Mertens, Anja Urbschat, Elisa Tomat, Eman A. Akam, Patrick Baer, Frederik C. Roos, Bernhard Brüne, Michaela Jung

**Affiliations:** 1 Institute of Biochemistry I, Goethe-University Frankfurt, Theodor-Stern-Kai 7, 60590 Frankfurt am Main, Germany; matthias.schnetz@t-online.de (M.S.); meier@biochem.uni-frankfurt.de (J.K.M.); rehwald@biochem.uni-frankfurt.de (C.R.); c.mertens86@gmail.com (C.M.); B.Bruene@biochem.uni-frankfurt.de (B.B.); 2 Institute for Biomedicine, Aarhus University, C. F. Møllers Allé 6, 8000 Aarhus, Denmark; anja.urbschat@staff.uni-marburg.de; 3 Department of Chemistry and Biochemistry, University of Arizona, 1306 E. University Blvd., Tucson, AZ 85721-0041, USA; tomat@email.arizona.edu (E.T.); EAKAM@mgh.harvard.edu (E.A.A.); 4 Division of Nephrology, Department of Internal Medicine III, Goethe-University Frankfurt, Theodor-Stern-Kai 7, 60590 Frankfurt am Main, Germany; p.baer@em.uni-frankfurt.de; 5 Clinic of Urology, Goethe-University Frankfurt, Theodor-Stern-Kai 7, 60590 Frankfurt am Main, Germany; Frederik.Roos@kgu.de; 6 German Cancer Consortium (DKTK), partner site Frankfurt/Mainz, 60590 Frankfurt am Main, Germany; 7 Frankfurt Cancer Institute, Goethe-University Frankfurt, 60596 Frankfurt am Main, Germany; 8 Project Group Translational Medicine and Pharmacology TMP, Fraunhofer Institute for Molecular Biology and Applied Ecology, 60596 Frankfurt am Main, Germany

**Keywords:** renal cell carcinoma, iron, macrophages, chelation therapy

## Abstract

Accumulating evidence suggests that iron homeostasis is disturbed in tumors. We aimed at clarifying the distribution of iron in renal cell carcinoma (RCC). Considering the pivotal role of macrophages for iron homeostasis and their association with poor clinical outcome, we investigated the role of macrophage-secreted iron for tumor progression by applying a novel chelation approach. We applied flow cytometry and multiplex-immunohistochemistry to detect iron-dependent markers and analyzed iron distribution with atomic absorption spectrometry in patients diagnosed with RCC. We further analyzed the functional significance of iron by applying a novel extracellular chelator using RCC cell lines as well as patient-derived primary cells. The expression of iron-regulated genes was significantly elevated in tumors compared to adjacent healthy tissue. Iron retention was detected in tumor cells, whereas tumor-associated macrophages showed an iron-release phenotype accompanied by enhanced expression of ferroportin. We found increased iron amounts in extracellular fluids, which in turn stimulated tumor cell proliferation and migration. In vitro, macrophage-derived iron showed pro-tumor functions, whereas application of an extracellular chelator blocked these effects. Our study provides new insights in iron distribution and iron-handling in RCC. Chelators that specifically scavenge iron in the extracellular space confirmed the importance of macrophage-secreted iron in promoting tumor growth.

## 1. Introduction

Iron is the most abundant transition metal in the human body and drives a variety of mechanisms considered as hallmarks of cancer. Due to its role as critical cofactor for the rate-limiting step of DNA synthesis, iron controls cell division, DNA repair, and chromatin remodeling [1]. Iron is essential for basic cellular processes such as mitochondrial respiration and the enhanced metabolic turnover under cancerous conditions is controlled by iron-sulfur cluster proteins [2]. Considering the poor bioavailability of iron and its potent role in tumorigenesis, the interplay of different proteins important for iron import, storage, and export has to be tightly regulated through the interplay of various proteins, including the major iron storage protein ferritin with its subunits ferritin light chain (FTL) and ferritin heavy chain (FTH), the iron exporter ferroportin (FPN), transferrin receptor 1 (TfR1) for iron uptake, and iron-regulatory proteins 1 and 2 (IRP1/2) [3]. 

The kidney plays a unique role in systemic iron homeostasis by filtering and reabsorbing iron as well as providing the main body source of erythropoietin, which promotes hemoglobin synthesis [4]. It was previously shown that renal iron overload in anemic patients requiring chronic transfusions enhanced the incidence of renal cell carcinoma (RCC) development [5]. Repeated injections of iron led to RCC development with increased metastasis to the lungs and lymph nodes in experimental models [6]. Recently, the expression of TfR1 was associated with progression and mortality in clear cell RCC (ccRCC), identifying TfR1 as a novel RCC biomarker and potential therapeutic target [7]. Despite these compelling observations and the fact that RCC is one of the 15 most common cancers in humans as well as the third most common cause of death among urological cancers in 2018 [8], the role of iron for renal cancer was not investigated in detail so far. As RCC is considered to be resistant against conventional chemo- and radiation therapy, medical therapeutic options are currently still limited, thus making nephrectomy the first treatment approach in localized disease [9]. For metastatic disease state, treatment options include systemic therapy with multitarget tyrosine kinase inhibitors (TKIs), including sunitinib, cabozantinib, and pazopanib as well as mammalian target of rapamycin (mTOR) inhibitors such as everolimus or temsirolimus, offering only modest benefits [10]. Novel promising approaches for the treatment of metastatic RCC include immunotherapy and immune checkpoint inhibitors (ICI) targeting the cytotoxic-T-lymphocyte-associated antigen 4 (CTLA-4) and programmed death-1 (PD-1) with monoclonal antibodies [11]. Herein, besides to monotherapy a combinatory immunotherapy with checkpoint inhibitors has recently been approved on the base of a clinical phase-3 trial [12]. However, it is clear that there is still an urgent need for a deeper understanding of the molecular processes underlying RCC, which could provide new strategies to interfere during cancer therapy or might help to better determine patient prognosis.

Based on its concentration-dependent toxicity under physiological conditions, cellular iron homeostasis has to be strictly regulated [13]. This balance is shown to be compromised in the tumor microenvironment [14]. The malignant state of cancer cells is associated with a deregulation in cellular iron homeostasis, particularly in the expression of iron-regulated genes to fuel their higher metabolic iron demand needed for division, growth, and survival [14]. Cancer cells of various tumor entities develop an iron retaining phenotype by upregulating FTL, FTH [15,16], TfR1 [7,17], and IRP1/2 [18], while downregulating the iron exporter FPN [17]. These alterations result in increased tumor growth, aggressiveness and a poor patient outcome [14,19]. However, it still remains partly unclear how cancer cells acquire iron from the tumor microenvironment. One of the key players of iron homeostasis are macrophages (MΦ), which play a dual, activation-dependent role in iron homeostasis [20]. While classical, pro-inflammatory MΦ sequester iron to restrict iron availability for bacterial growth [21], alternatively activated anti-inflammatory MΦ recycle iron from dying cells by enhanced phagocytic activity [22]. Due to their physiological function, alternatively activated MΦ promote tissue repair, cell proliferation, and angiogenesis [23]. In the context of carcinogenesis, tumor associated MΦ (TAM) are major players when looking at abundance [24] and pro-tumoral function [25]. TAMs show characteristics of both pro-inflammatory MΦ that create an inflammatory environment during early stages of tumor development as well as anti-inflammatory MΦ [25,26] during later stages that suppress anti-tumor immunity and stimulate tumor neovascularization as well as metastasis [27,28]. Accordingly, TAMs were shown to positively associate with tumor progression and worse patient prognosis [29,30,31]. 

Although the control of iron availability in the tumor microenvironment seems to be crucial for tumor development, the distribution of iron within cellular compartments of the tumor, in particular tumor cells and TAMs, as well their association with tumor outcome have not been investigated so far in renal cancer. In the present study, we provide evidence that iron-dependent genes are highly expressed in renal cancer and are associated with tumor pT-stage (tumor size and invasion as defined by UICC) and tumor grade. We further show that TAMs adopt an iron-release phenotype with increased expression of the iron exporter FPN, whereas tumor cells retain intracellular iron. *In vitro* assays with patient-derived extracellular fluids as well as novel extracellular iron chelators showed the iron-dependence of renal tumor growth and metastasis.

## 2. Results

### 2.1. Iron Homeostasis Is Altered in RCC

In order to determine whether renal iron homeostasis is altered in RCC, we first analyzed mRNA expression of several iron-dependent genes, including *FPN*, *FTL*, *FTH*, *IRP2,* and *TfR1* in whole tissue homogenates of our patient cohort (Table 1). 

**Figure 1:**
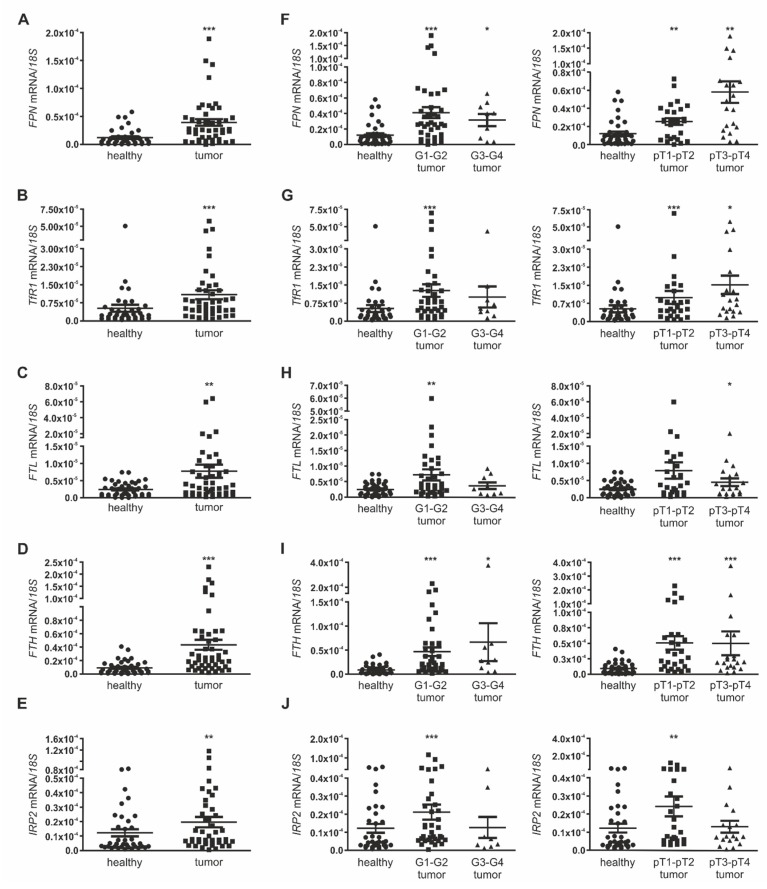
Expression of iron-regulated genes in human renal cancer samples. mRNA expression normalized to the housekeeping gene *18S* in whole tissue homogenates of renal tumor tissue and adjacent healthy tissue of (**A**) *FPN* (*n* = 48), (**B**) *TfR1* (*n* = 47), (**C**) *FTL* (*n* = 48), (**D**) *FTH* (*n* = 48), and (**E**) *IRP2* (*n* = 46). (**F**–**J**) Left: mRNA expression of (**F**) *FPN*, (**G**) *TfR1*, (**H**) *FTL*, (**I**) *FTH,* and (**J**) *IRP2* correlated to low (G1-G2) and high (G3-G4) tumor grade. Right: mRNA expression of (**F**) *FPN*, (**G**) *TfR1*, (**H**) *FTL*, (**I**) *FTH,* and (**J**) *IRP2* correlated to low (pT1–pT2) and high (pT3–pT4) tumor pT-stage. Number of tested patients differ between genes due to patients with failed measurements of initially low sample RNA amount. No samples have been excluded as outliers. Graphs are displayed as means ± SEM with * *p* < 0.05, ** *p* < 0.01, *** *p* < 0.001.

We found a significantly increased mRNA expression in tumor tissue compared to adjacent healthy tissue for all genes (Figure 1A–E). We performed hematoxylin staining in both healthy adjacent tissue and RCC subtypes of clear cell RCC (ccRCC), papillary RCC (pRCC) as well as chromophobe RCC (chRCC) that were included in our patient cohort (Figure S1A), and analyzed the *CAIX* mRNA expression, which was shown to be upregulated in more than 90% of RCC cases [32] (Figure S1B). Accordingly, *CAIX* mRNA expression was significantly upregulated in ccRCC and pRCC tumor subtypes, whilst varying in chRCC compared to adjacent healthy tissue. We next analyzed the mRNA expression of iron-dependent genes in relation to tumor grade (G1-G2 vs. G3-G4) and tumor pT-stage (pT1 pT2 vs. pT3-pT4). *FPN* mRNA expression was significantly increased in all tumor pT-stages and tumor grades compared to adjacent healthy tissue with the notion of enhanced expression in higher tumor pT-stage (Figure 1F). This expression pattern was also observed for mRNA expression of *TfR1* (Figure 1G). 

For *FTL*, *FTH,* and *IRP2,* we found an increased mRNA expression in lower tumor grades (G1-G2) and lower tumor pT-stage (pT1–pT2), but either similar or lower expression within the group of higher tumor grades (G3-G4) and higher tumor pT-stage (pT3–pT4; Figure 1H–J). 

**Figure 2:**
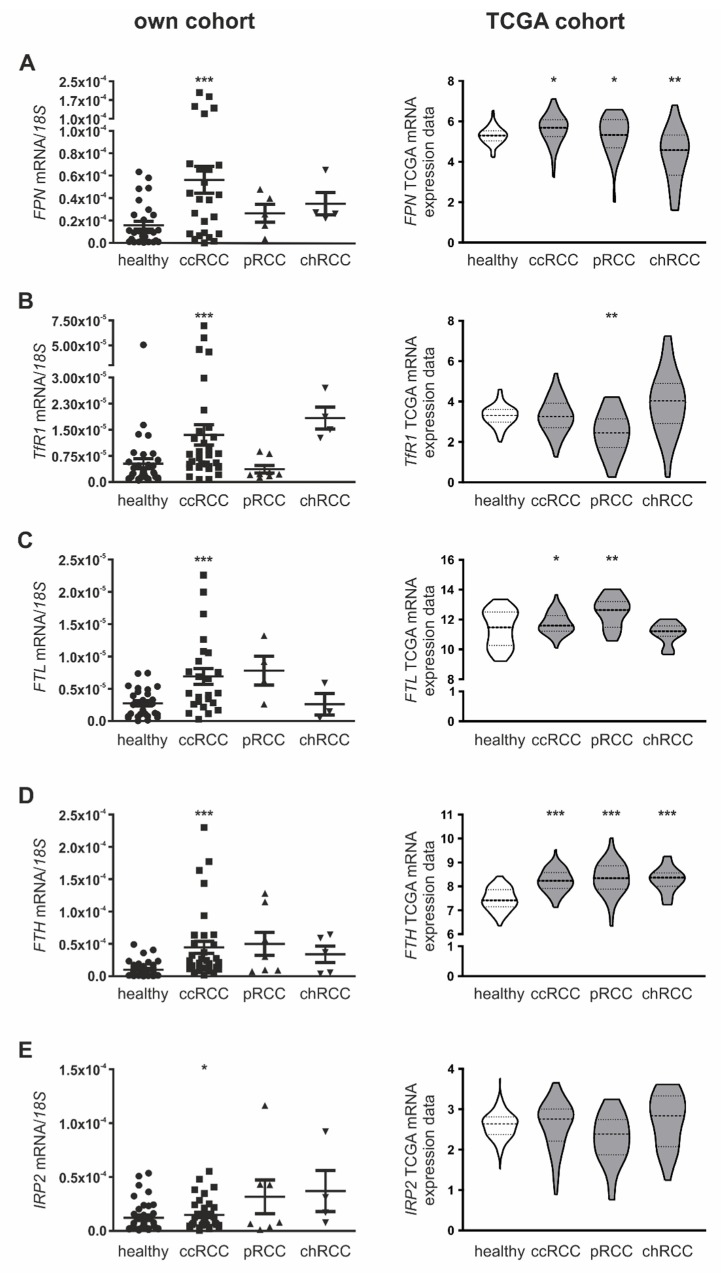
Profile of iron-regulated genes in histopathologically distinct RCC subtypes. mRNA expression of renal tumor and adjacent healthy samples in clear cell (ccRCC), papillary (pRCC), and chromophobe (chRCC) RCC of own patient cohort (left) compared to mRNA expression acquired from the TCGA database applying the ccRCC-KIRC (*n* = 70), pRCC-KIRP (*n* = 31), and chRCC-KICH (*n* = 23) datasets (right). Analyzed genes include (**A**) *FPN*, (**B**) *TfR1*, (**C**) *FTL*, (**D**) *FTH,* and (**E**) *IRP2*. Own cohort is normalized to housekeeping gene *18S* expression. Graphs are displayed as means ± SEM with * *p* < 0.05, ** *p* < 0.01, *** *p* < 0.001.

Since RCC subtypes significantly differ regarding in the prognosis and treatment [33], we analyzed the mRNA expression of iron-dependent gene expression in patients with ccRCC, pRCC, or chRCC of our cohort (Figure 2A–E, left panel). While the defined iron-dependent genes were significantly upregulated within the ccRCC subgroup in comparison to adjacent healthy tissue, mRNA expression in the pRCC and the chRCC subtype varied, depending on the analyzed gene. Expression of *FPN*, *FTH*, and *IRP2* was higher in all RCC subtypes compared to adjacent healthy tissue, whereas *FTL* remained unaltered in the chRCC subtype and *TfR1* was lower in pRCC subtypes. In order to verify our data, especially regarding patients diagnosed with pRCC and chRCC, where less patients were included in our cohort, we analyzed publically available TCGA KIRC (ccRCC), KIRP (pRCC), and KICH (chRCC) data sets (Figure 2A–E, right panel). RNA expression in the TCGA data sets confirmed a significant upregulation of *FPN*, *FTL*, and *FTH* in ccRCC. In pRCC, *FPN*, *FTL,* and *FTH* are significantly higher expressed, while *IRP2* remained unaltered. Our data regarding reduced *TfR1* expression in pRCC and unaltered *FTL* expression in chRCC was corroborated using the TCGA data analysis.

**Figure 3:**
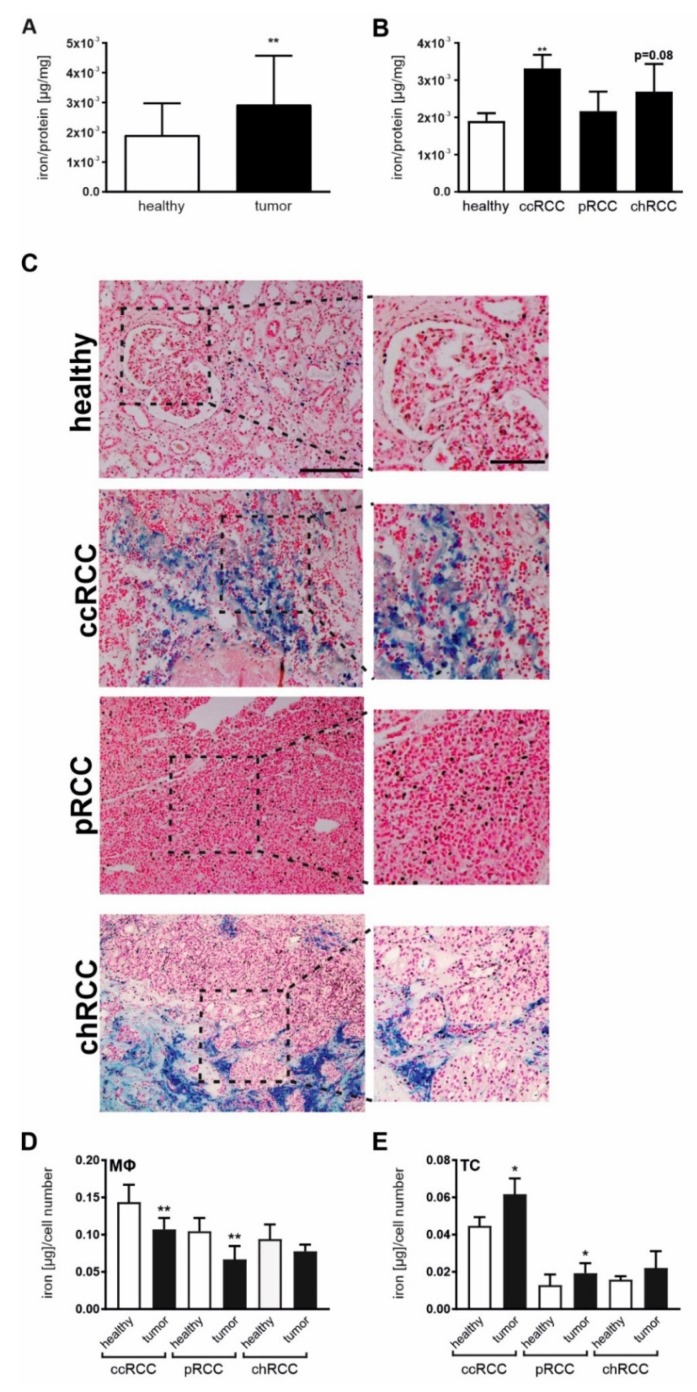
Iron homeostasis and distribution is altered in RCC. (**A**) Iron load normalized to protein amount in whole tissue homogenates of renal cancer tissue in comparison to adjacent healthy renal tissue measured by AAS (*n* = 31). (**B**) Iron load in whole tissue homogenates of clear cell (ccRCC; *n* = 17), papillary (pRCC; *n* = 7), and chromophobe (chRCC; *n* = 7) RCC in comparison to corresponding healthy renal tissue measured by AAS. (**C**) Representative pictures of Perl’s staining of RCC tissue and adjacent healthy renal tissue of ccRCC, pRCC, and chRCC. Representative pictures (scale bar: 200 µm) with corresponding detailed pictures (scale bar: 100 µm) are given. (**D,E**) Macrophages (MΦ) and CD326+ cells were isolated by FACS-sorting from RCC tissue and adjacent healthy tissue. Intracellular iron load of (**D**) MΦ and (**E**) either tumor cells (TC) or epithelial cells from adjacent healthy tissue of ccRCC (*n* = 7), pRCC (*n* = 13), and chRCC (*n* = 4) measured by AAS. Statistical analysis was performed comparing tumor to adjacent healthy tissue within the histopathological subtypes. Graphs are displayed as means ± SEM. * *p* < 0.05, ** *p* < 0.01.

As we showed an altered iron homeostasis in all histopathological subtypes, we next aimed at looking into the iron distribution in RCC tissue. We first analyzed the iron amount of tumor and adjacent healthy tissues by AAS measurements. Tumor tissue showed an overall significantly higher iron amount than adjacent healthy renal tissue (Figure 3A). When analyzing the histopathological subtypes, both ccRCC and chRCC showed a higher iron amount compared to adjacent healthy tissue, whereas in pRCC the total iron amount remained nearly unaltered (Figure 3B). To address the question of iron localization within the tissues, Perl’s staining of tumor versus adjacent healthy tissue slides was used. In line with our AAS analysis, healthy renal tissue showed a low amount of iron deposits appearing in blue. Compared to the healthy adjacent tissue, a more intense staining in ccRCC was observed, whereas iron deposits in pRCC remained low (Figure 3C and Figure S2A–D). Intriguingly, the iron load in chRCC varies considerably between different patients (Figure S2D) with the notion of overall enhanced iron deposits in tumor tissue compared to adjacent healthy tissue. In ccRCC tissue, we hypothesize that the highly intense blue-colored cells might be tumor cells, whereas the diffuse positive staining around long-shaped cells in the stroma might be iron secreted by MΦ. There are also other positive-stained cells in the stroma that appear much smaller, which we believe might be lymphocytes that are also able to handle iron in the tumor stroma as previously described by Marques et al. in mammary carcinoma [34]. For pRCC we only detect low amounts of overall Perl’s staining, with localized positive staining mostly in tumor cells, whereas we observed high amounts of iron deposits in chRCC, mostly within the tumor stroma. We and others previously showed that tumor cells are prone to adopt an iron retaining phenotype, whereas cells from the tumor stroma such as MΦ rather adopt an iron mobilization and iron releasing phenotype [34,35]. In order to verify the location of iron within different tumor compartments in RCC tissues, we sorted both tumor cells and tumor-associated MΦ from tumor tissue of all histopathological RCC subtypes and compared them to sorted epithelial cells and MΦ isolated from adjacent healthy tissue (Figure 3D,E). A significantly reduced intracellular iron amount in MΦ isolated from ccRCC and pRCC tissues was observed, whereas MΦ from chRCC tissues showed similar intracellular iron levels as cells from adjacent healthy tissue (Figure 3D). In contrast, tumor cells showed a significant increased iron amount in ccRCC and pRCC compared to adjacent renal epithelial cells. In chRCC, iron amount in tumor cells showed a larger variation resulting in a non-significant increase compared to renal epithelial cells isolated from adjacent healthy tissue (Figure 3E). 

### 2.2. Iron Promotes Renal Tumor Cell Growth

**Figure 4:**
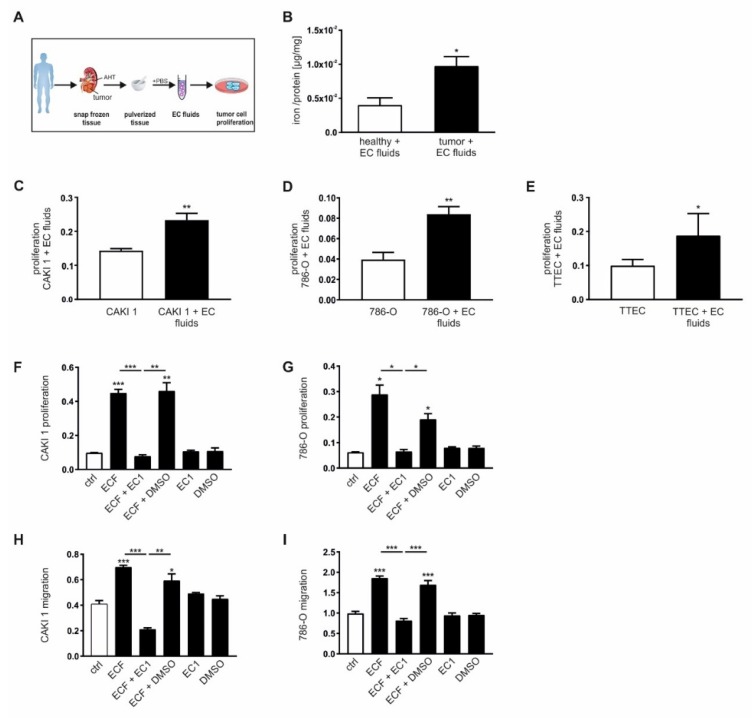
Extracellular iron induces proliferation and migration of tumor cells in vitro. (**A**) Schematic overview of how to generate extracellular (EC) fluids (ECF) from primary human renal tumor and adjacent healthy tissue. (**B**) Iron load measured by AAS relative to the total protein amount of EC fluids of ccRCC tissue compared to adjacent healthy renal tissue (*n* = 8). Proliferation of (**C**) CAKI-1 (*n* = 7), (**D**) 786-O (*n* = 8), and (**E**) primary human tumor tubular epithelial cells (TTEC) upon stimulation with EC fluids in vitro measured with the xCELLigence system (*n* = 8). Proliferation of **(F)** CAKI 1 (*n* = 4) and (**G**) 786-O (*n* = 4) cells as well as migration of (**H**) CAKI 1 (*n* = 4) and (**I**) 786-O cells (*n* = 4) upon stimulation with EC fluids in the presence or absence of an extracellular chelator (EC1, 100µM) or dimethyl sulfoxide (DMSO) as negative control measured with the xCELLigence system. Graphs are displayed as means ± SEM with * *p* < 0.05, ** *p* < 0.01, *** *p* < 0.001.

In order to test the role of iron released into the tumor stroma, we generated extracellular fluids (EC fluids) from both tumor tissue as well as adjacent healthy tissue (Figure 4A). First, we analyzed the iron amount in EC fluids by AAS and observed significantly higher iron amounts in EC fluids isolated from tumor tissue as compared to EC fluids from adjacent healthy tissue (Figure 4B). We then stimulated renal tumor cells CAKI-1 (Figure 4C) and 786-O (Figure 4D) as well as primary patient-derived tumor tubular epithelial cells (TTEC; Figure 4E) with tumor EC fluids. Cellular proliferation was analyzed applying xCELLigence real-time measurements. Results showed that all tested cell lines as well as primary tumor cells positively responded to treatments with tumor EC fluids and augmented cellular proliferation upon stimulation. 

To further verify the role of extracellular iron on tumor proliferation and migration, we stimulated tumor cells with EC fluids in the presence of a specific extracellular chelator (EC1). This novel compound was designed for extracellular chelation as it features an established iron-binding unit as well as a negatively charged group to hinder cell membrane permeation (Figure S3A). In particular, the tridentate chelating unit of EC1 includes a thiosemicarbazone moiety that is common to many anti-proliferative iron chelators [36,37]; however, the incorporation of a negatively charged sulfonate group significantly limits the ability of EC1 to cross cellular membranes. As a result, EC1 is expected to chelate iron only in the extracellular space without affecting intracellular iron levels. The iron binding abilities were validated using optical absorption spectroscopy (Figure S3B). The effects of EC1 on cellular viability and proliferation were tested in vitro in CAKI-1 and 786-O cells in comparison to the unspecific chelator 2,2′-dipyridine (2′2-DPD) and the intracellularly activated prochelator (TC3-S)_2_ [38,39,40]. Whereas EC1 showed no effect at concentrations up to 100 µM with regard to both, viability (Figure S3C,D) and cellular proliferation (Figure S3E,F) under basal growth conditions, both 2′2-DPD and (TC3-S)_2_ showed increasingly adverse effects at higher concentrations regarding cellular viability and anti-proliferative capacity due to the fact that both are able to chelate intracellular iron. In contrast, EC1 showed toxicity effects only at very high concentrations (500 µM), which might be due to non-specific side effects. Supplementation of EC fluids with EC1 (100 µM) in order to specifically block iron secreted to the supernatant resulted in a significant inhibition of cellular proliferation and migration of both CAKI-1 (Figure 4F,H) and 786-O cells (Figure 4G,I).

### 2.3. Tumor Proliferation by Macrophage-Secreted Iron Is Suppressed by EC1

**Figure 5:**
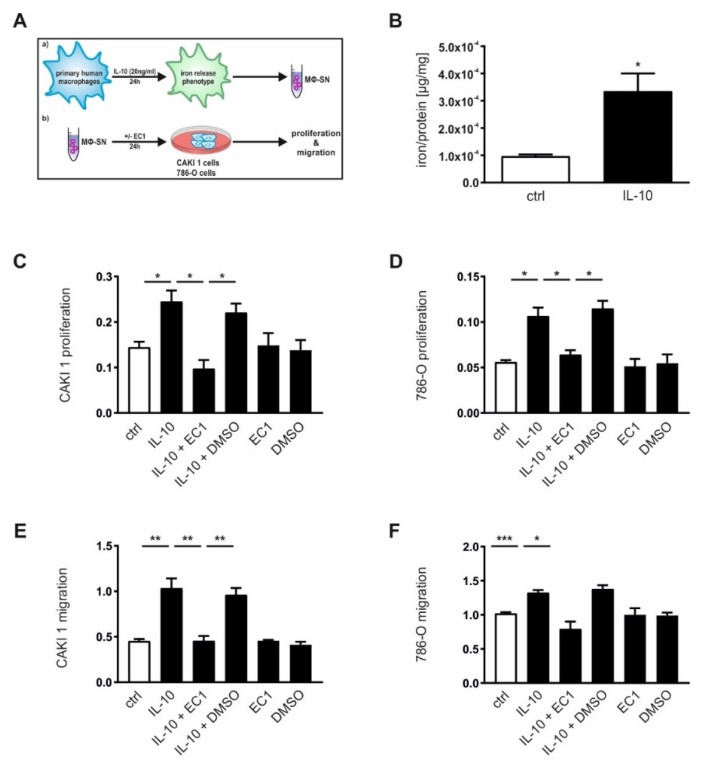
Macrophage-secreted iron induces proliferation and migration of tumor cells in vitro. (**A**) Schematic overview of how to generate conditioned medium from iron-releasing human MΦ. (**B**) Iron amount measured by AAS relative to the total protein amount in the supernatant of primary human MΦ, either left untreated (ctrl) or stimulated with IL-10 (20 ng/mL; 24 h) (*n* = 5). Proliferation of (**C**) CAKI 1 (*n* = 4) and (**D**) 786-O (*n* = 4) cells as well as migration of **(E)** CAKI 1 (*n* = 4) and (**F**) 786-O cells (*n* = 4) upon stimulation with the supernatant of IL-10-stimulated MΦ in the presence or absence of an extracellular chelator (EC1, 100 µM) or dimethyl sulfoxide (DMSO) as negative control measured with the xCELLigence system. Graphs are displayed as means ± SEM with * *p* < 0.05, ** *p* < 0.01, *** *p* < 0.001.

According to our previous observation that tumor-associated MΦ adopt an iron releasing phenotype (Figure 3D), we further established an in vitro setting to analyze the role of macrophage-secreted iron in conferring renal tumor cell growth (Figure 5A). 

As previously published [41,42], IL-10 stimulation of primary human MΦ induced the release of iron into the supernatant measured by AAS (Figure 5B). We next applied macrophage-conditioned supernatants to renal tumor cells CAKI-1 (Figure 5C,E) and 786-O (Figure 5D,F) and observed enhanced proliferation (Figure 5C,D) as well as tumor cell migration (Figure 5E,F) measured by xCELLigence in real-time upon stimulation with IL-10-conditioned media. 

To further verify the effect of EC1 on tumor proliferation and migration in the presence of macrophage-secreted iron, we stimulated tumor cells with MΦ-conditioned media supplemented by EC1 (100 µM). In line with our previous observations using EC fluids (Figure 4F–I), EC1 was able to significantly inhibit cellular proliferation and migrations of both CAKI-1 (Figure 5C,E) and 786-O cells (Figure 5D,F).

### 2.4. Macrophage-Derived Iron Is Exported Via the Iron Exporter FPN, Which Is Positively Associated with Poor Patient Outcome

**Figure 6:**
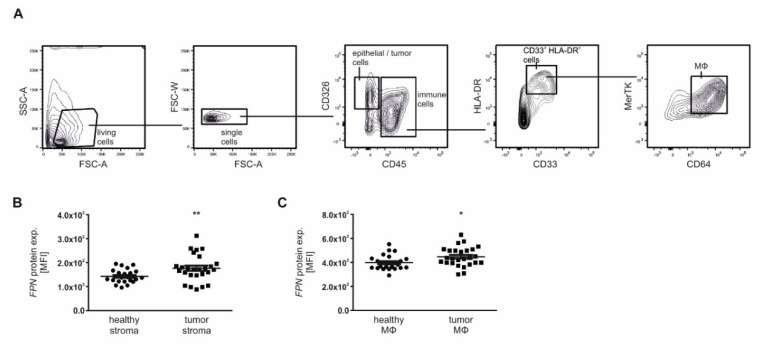

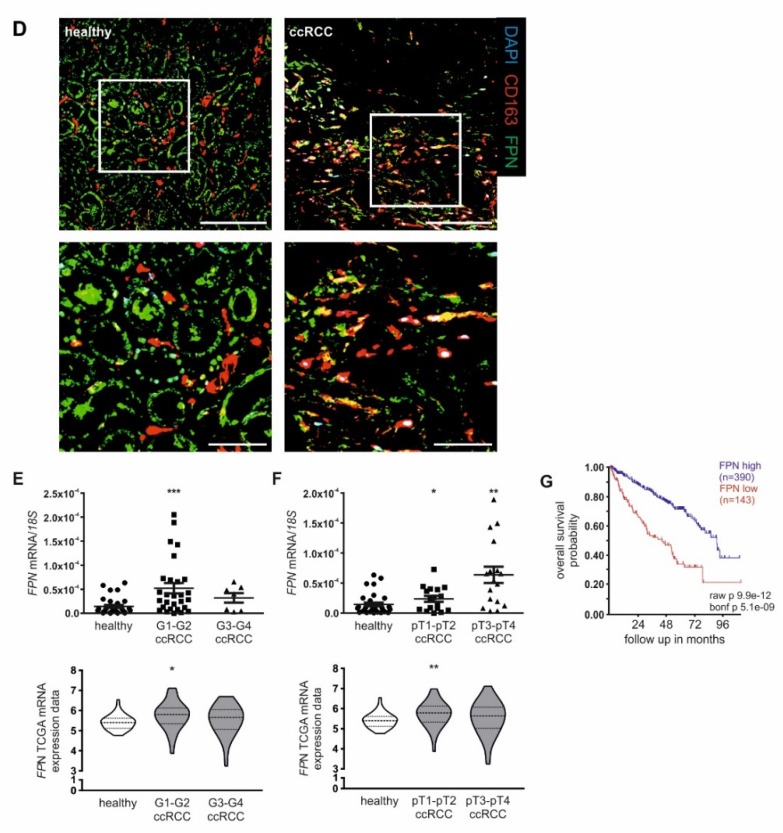
Tumor-associated MΦ express enhanced FPN protein. (**A**) FACS panel how to gate CD45^+^ immune cells and CD326^+^ epithelial/tumor cells. Immune cells were further gated for CD33^+^/HLA-DR^+^ cells of which CD64^+^ and MerTK^+^ MΦ were sub-selected. Cells were subsequently analyzed for their FPN protein expression, displayed as MFI (mean fluorescence intensity). (**B**) FPN protein expression as MFI of CD45^-^/CD326^-^ stroma cells (*n* = 26). (**C**) FPN protein expression as MFI of MΦ in tumor tissue compared to adjacent healthy tissue (*n* = 26). (**D**) Representative pictures for the MΦ marker CD163 and FPN protein expression in tumor tissue compared to healthy adjacent tissue applying confocal laser scanning miscroscopy. DAPI was used as nuclear stain. Scale bar: 200 µm. *FPN* mRNA expression normalized to *18S* expression correlated to (**E**) low (G1–G2) and high (G3–G4) tumor grade and (**F**) low (pT1–pT2) and high (pT3–pT4) tumor pT-stage in our patient cohort (upper panels; *n* = 48) compared to the TCGA data base, applying the ccRCC-KIRC data set (lower panels; *n* = 70). **(G)** Kaplan-Meier curve of high or low *FPN* expression from the R2 bioinformatics platform using the ccRCC-KIRC data set. Graphs are displayed as means ± SEM with * *p* < 0.05, ** *p* < 0.01, *** *p* < 0.001.

We next asked whether the iron exporter FPN was expressed in tumor-associated MΦ. Therefore, FPN protein expression in MΦ by flow cytometry (Figure 6A) was measured, showing higher FPN expression in both tumor stroma (Figure 6B) as well as tumor-associated MΦ (Figure 6C) compared to stroma and MΦ from adjacent healthy tissue. In order to localize FPN protein within the tissue, multiplex-immunohistochemistry was applied, combining CD163 as macrophage marker, FPN, and DAPI as nuclear stain (Figure 6D), showing enhanced co-localization of CD163 and FPN in tumor tissue compared to adjacent healthy tissue. Taking our previous *FPN* mRNA data into consideration that showed enhanced *FPN* expression in tumor tissue compared to adjacent healthy tissue (Figure 1A and Figure 2A), we next questioned the association of *FPN* expression with tumor grade (Figure 6E) and tumor (Figure 6F) of our own cohort (upper part) compared to the TCGA data set (lower part). In line with the TCGA data set, we observed association of FPN mRNA expression only with lower tumor grade (Figure 6E). For tumor pT-stage (Figure 6F), a positive association with lower tumor pT-stages (pT1–pT2) was noticed, which was more pronounced in higher tumor pT-stages (pT3–pT4). However, TCGA data suggests higher FPN expression in patients with low tumor grade (G1–G2) and tumor pT-stage (pT1–pT2). Accordingly, low tissue FPN expression correlated with a lower overall survival probability analyzed by the R2: Genomics Analysis and Visualization Platform applying the ‘Tumor Kidney Renal Clear Cell Carcinoma—TCGA-533′ data set (Figure 6G).

## 3. Discussion

We present evidence that iron metabolism is significantly altered in renal cancer. We observed elevated iron deposits in renal tumor tissue compared to adjacent healthy tissue as well as enhanced expression of iron-regulated genes in tumor tissue isolated from patients with renal cancer compared to adjacent healthy tissue. As iron is of importance for essentially all tumor hallmarks, we further investigated the role of iron in determining tumor cell proliferation and migration. In this setting, we also described and characterized the use of a novel extracellular iron chelator that scavenges iron in the extracellular space, thereby providing a valuable tool to investigate its role in the tumor microenvironment. 

Numerous studies support a positive association between increased iron levels and cancer development, whereby cancer cells evolved specialized mechanisms for iron acquisition, storage, and mobilization in order to ensure their enhanced metabolic turnover [43]. Despite the fact that both availability as well as distribution of iron is strictly regulated under healthy conditions, cancers exert a profoundly dysregulated iron-handling capacity with altered expression of iron-regulated genes [15,16,17,44,45]. These observations are corroborated by the present study, detecting enhanced expression of iron-regulated genes in renal tumor tissue as compared to adjacent healthy tissue, which was most prominent in ccRCC. This effect was further confirmed by TCGA data base analysis. In this regard, we also found that cancer cells isolated from patients with renal cancer showed enhanced iron sequestration compared to their healthy counterparts. 

Taking the unique role of the kidney in iron physiology into consideration [4], several markers, including erythropoietin [46] have been tested in RCC. Regardless of the initial promising effects, they only showed low predictive value. There are still no specific and reliable tumor markers neither for RCC diagnosis nor for monitoring post-operative disease outcome. Despite the apparent association of RCC with the development of systemic anemia in RCC patients [47], the role of iron in human RCC carcinogenesis is largely unknown and was only scarcely investigated so far. Because of the growing evidence on their tumor-promoting effects, the expression of iron-regulated genes could become an important factor among the markers of tumorigenesis. Along these lines, Greene at al. recently showed an association of TfR1 expression and RCC progression [7], with TfR1 levels being highest in benign primary tumors, subsequently dropping during the course of disease progression. TfR1 levels were therefore inversely associated with worse survival, but independent of tumor pathology. In line, we observed overall enhanced iron amounts in tumor tissue as compared to adjacent healthy tissue for all investigated renal cancer subtypes. Interestingly, iron amounts in chRCC varied considerably between different patients and needs to be further addressed in follow-up studies including higher patient number. Intriguingly, we also found initial differences in iron levels in adjacent healthy tissue for each histopathological subtype. These observations might arise both from the original localization of the tissue for individual samples as well as result from different basal iron levels of each individual patient. Therefore, healthy control tissue has to be controlled carefully to avoid misleading interpretation. 

Nonetheless, modulating the iron-retaining tumor phenotype reduced growth and progression of both human and mouse carcinomas [48]. The use of iron chelators in the treatment of cancer inhibited DNA synthesis and caused a G1-S-phase cell cycle arrest, attenuated epithelial-mesenchymal-transition, and promoted cancer cell apoptosis [39]. Furthermore, chemically-induced and oncogene-driven cancer models corroborated these findings and stressed the relevance of iron for tumor development [49,50]. Numerous studies investigated methods to interfere with iron-handling in cancer cells, either by directly modulating iron-regulated genes [43] or by the use of iron chelators [37]. Nevertheless, a detailed knowledge of the effects of chelators within the tumor microenvironment (and on potential iron sources thereof) is still lacking [43]. In this study, we identified enhanced iron levels in extracellular fluids of tumor tissue in comparison to healthy adjacent tissue, suggesting that cells of the tumor microenvironment secreted iron in extracellular fluids. Due to their important role in tumor development and iron handling, we proposed that MΦ might adopt a pro-tumorigenic iron-releasing phenotype, whereby tumor growth is favored. Since MΦ are central players in systemic iron homeostasis, they have evolved unique mechanisms to recycle, store, and release iron to their local microenvironment. However, our data suggest differences in total tissue iron levels versus iron amounts of both MΦ and tumor cells for histopathological renal cancer subtypes. We hypothesize that even if the overall amount of iron is not changed in tumor tissue compared to adjacent healthy tissue in pRCC patients, the different distribution of iron within the tissue and in cells of the tumor mass, i.e. MΦ or tumor cells might add to the characteristics of iron as a pro-tumoral factor. 

Macrophage iron homeostasis is functionally coupled to their heterogeneity and plasticity, with their polarization status being reflected also by their expression profiles of iron-regulated genes. We previously showed that treatment of MΦ with LPS/IFNγ enhanced the retention of iron within the cell, whereas stimulation with anti-inflammatory cytokines such as IL-10 or IL-4 induced the release of iron [41]. This observation falls in line with a typical cytokine/chemokine profile of differentially polarized MΦ. We used this setting also in the present study to generate supernatant from IL-10-polarized iron-releasing MΦ. We tested the effect of iron, which was released by MΦ, in combination with an iron chelator that specifically binds iron in the extracellular space. EC1, which was designed and synthesized specifically for this study, is a thiosemicarbazone chelator featuring a sulfonate group that is negatively charged near neutral pH. While the tridentate (*O,N,S*) binding unit (see Figure S3A) ensures high-affinity iron coordination, the negative charge on the scaffold was incorporated to limit or hamper cellular membrane permeability. This strategy is particularly advantageous for the study of iron with respect to the crosstalk between TAMs and cancer cells. We found that the addition of EC1 reversed the positive effect of macrophage-conditioned media on the proliferation and migration of cancer cells. Although the exact molecular speciation of iron released in the macrophage supernatant remains to be determined, these experiments indicated that this iron pool is accessible by small-molecule chelators and could represent a hitherto unrecognized effect of these antiproliferative compounds in the tumor microenvironment.

Our previous studies using intracellularly active pro-chelators underscore the importance of macrophage-released iron for tumor cell proliferation [41]. Intriguingly, current research focuses primarily on the role of iron and iron-chelation therapy in tumor cells, whereas detailed knowledge on the crosstalk between tumor cells and tumor-associated MΦ as a possible source of iron is lacking. Taking into account that the presence of MΦ in tumor tissue is closely linked to tumor progression, we further analyzed the expression of the iron exporter FPN as a determinant of the iron releasing capacity of MΦ. We found enhanced expression of FPN in tumor MΦ compared to MΦ of adjacent healthy tissues, which was significantly associated with tumor pT-stage. However, these data could not be corroborated by TCGA data base analysis regarding overall patient survival. This discrepancy might arise from low cohort size of analyzed patients. Furthermore, it might also be necessary to distinguish FPN expression in stromal cells versus tumor cells as compared to whole tissue analysis. Recently, it was shown that tumor-associated MΦ also secrete iron in form of FT, which, in turn, stimulated tumor cell proliferation [44]. FTL expression in MΦ was further described as an independent prognostic marker in node-negative breast cancer. However, in the present study, we did not observe significant changes in FT protein expression in tumor-associated MΦ compared to MΦ isolated from adjacent healthy tissue. Recently, Marques et al. observed that FT expression was elevated in tumor-infiltrating lymphocytes, whereas no changes were detected for FT expression in tumor-associated MΦ [34]. The crucial implication of MΦ in tumor development and their role in iron distribution within the tumor microenvironment represents an important area of investigation in contemporary cancer biology.

Collectively, the results of the present study indicate that iron homeostasis is significantly disturbed in renal cancer with most of the investigated iron-regulated genes being associated with tumor grade and tumor pT-stage. Moreover, we observed that iron availability in the tumor microenvironment might be controlled by tumor-associated MΦ, which adopt an iron-release phenotype through increased expression of FPN. Application of chelators that are able to specifically scavenge iron in the extracellular space confirmed the importance of macrophage-secreted iron in promoting tumor cell proliferation and migration. 

Future experimental in vivo studies should address the possibility to either interfere with iron availability in the tumor microenvironment or use macrophage-targeted chelation strategies. Moreover, more research is needed with regard to the questions of: i) the molecular networks that allow tumor cells to actually take up, store, and utilize iron and ii) the release of tumor cell-derived mediators that re-program stromal cells, i.e., MΦ, to serve as an iron source in order to maintain their enhanced metabolism and growth.

## 4. Materials and Methods 

### 4.1. Ethics

Investigations were conducted in accordance with the ethical standards according to the Declaration of Helsinki and to national and international guidelines. Primary human tumor and adjacent healthy tissues were obtained from 64 patients with the approval of the ethics committees of the Goethe-University Hospital Frankfurt am Main (04/09 UGO 03/10) and the Philipps-University Hospital Marburg (122/14). Patients gave their written informed consent prior to surgery (UCT 122/14 and 04/09 UGO 03/10).

### 4.2. Participants

Patients included in this study underwent nephrectomy or partial nephrectomy for renal lesions histopathologically diagnosed with renal cancer between 2016 and 2019 at University Hospitals Frankfurt am Main and Marburg (see Table 1). Patients underwent preoperative staging either by computed tomography or Magnetic resonance imaging and surgery was performed before receiving other therapy. Tissue was collected immediately after surgery and processed for single cell suspensions, fixed in 4% paraformaldehyde (PFA) or stored at −80 °C. Pathological examination was performed by independent pathologists applying the UICC TNM classification of malignant tumors [51].

### 4.3. RNA Extraction and Quantitative Real-Time PCR (qRT-PCR)

Total RNA was extracted from homogenized tissue samples using peqGold RNAPure (VWR, Darmstadt, Germany, 732-3312) and transcribed using Maxima First Strand cDNA synthesis kit (Thermo Fisher, Dreieich, Germany, K1642). Gene expression profiles were determined by qPCR using the SYBR Green Supermix (Bio-Rad, Munich, Germany, 1725006CUST) on a CFX-Connect real-time-PCR detection system (Bio-Rad). Results were quantified using the Bio-Rad CFX-Manager (Bio-Rad, version 3) with 18S mRNA expression as housekeeping control. All primers except TfR1 Primer (Qiagen, Hilden, Germany, QT00094850) are listed in the supplemental information file and were purchased from Biomers (Ulm, Germany).

### 4.4. Data baSe Analysis

To show mRNA expression of *FPN*, *FTL*, *FTH*, *TfR1* and *IRP2* in different renal cancer subtypes, gene expression data of the Cancer Genome Atlas were analyzed (https://portal.gdc.cancer.gov/). Expression data of TCGA files were used of the following data sets: “Tumor Kidney Renal Clear Cell Carcinoma” (KIRC, *n* = 533), “Tumor Kidney Renal Papillary Cell Carcinoma” (KIRP, *n* = 290), and “Tumor Kidney Chromophobe” (KICH, *n* = 66). Cases with tumor and adjacent renal healthy tissue data available were included in the analysis (KIRC: *n* = 70; KIRP: *n* = 31; KICH: *n* = 23).

Kaplan-Meier plots were generated using the R2 Genomics Analysis and Visualization Platform (http://r2.amc.nl). The dataset “Tumor Kidney Renal Clear Cell Carcinoma” (*n* = 533) was chosen. Default settings of the KaplanScan including a log rank comparison between the groups were used to determine an optimum survival cut-off as described in the portal. The resulting p-value as well as the Bonferroni correction of the log rank comparison are included in the plots.

### 4.5. Atomic Absorption Spectroscopy (AAS)

Iron measurements were performed as previously described [35]. Whole tissue homogenates where either measured as whole homogenates and normalized to total protein amount or underwent FACS sorting with the final cell suspension being analyzed for its iron content and normalized to the total number of sorted cells.

### 4.6. Perl’s Stain

Tissue slides were dewaxed in xylene and rehydrated in a series of alcohol solutions using decreasing concentrations. Perl’s stain was performed using the Iron Stain Kit (Sigma Aldrich, Taufkirchen, Germany, HT20) according to the manufacturer’s protocol. Slides were then washed in distilled water, counterstained with Nuclear Fast Red solution (Sigma Aldrich, N3020), rapidly dehydrated, and mounted in Entellan (Merck, Darmstadt, Germany, 107961). Pictures were acquired using an Axioskop 40 (Zeiss, Oberkochen, Germany).

### 4.7. Flow Cytometric Analyses

Tumors and adjacent healthy renal tissues were dissociated using the human Tumor Dissociation Kit (Miltenyi Biotec, Bergisch-Gladbach, Germany, 130-095-929) and GentleMACS System (Miltenyi Biotec). Samples were acquired with a LSRII/Fortessa flow cytometer (BD, Heidelberg, Germany) expressed as mean fluorescence intensity (MFI). CompBeads (BD) were used for single color compensation to create multi-color compensation matrices. For gating, fluorescence minus one (FMO) controls were used. Prior to experiments, all antibodies and secondary reagents were titrated to determine optimal concentrations.

For staining of FPN, extracellular staining of patient-derived single cell suspensions was performed, containing CD33 BV510 (BD, 563257), MerTK BV421 (Biolegend, San Diego, CA, USA, 367603), CD45 AF700 (Biolegend, 368513), CD 64 BV605 (Biolegend, 305033), CD206 PE-Cy7 (Biolegend, 321124), CD326 PE-CF594 (BD, 565399), HLA-DR APC-Cy7 (Biolegend, 307658), and FPN PE (Novus, Wiesbaden, Germany, NBP1-21502). 

### 4.8. FACS Sorting and Processing of Sorted Cells

Single cell suspensions of tumor and adjacent healthy renal tissue were stained with an antibody cocktail containing CD33 BV510 (BD, 563257), MerTK BV421 (Biolegend, 367603), CD45 AF700 (Biolegend, 368513), CD64 BV605 (Biolegend, 305033), CD326 PE-CF594 (BD, 565399), HLA-DR APC-Cy7 (Biolegend, 307658). Cell suspensions were sorted using a FACS Aria (BD) FACS sorter, resulting in CD45^−^/CD326^+^ epithelial cells and CD45^+^/CD33^+^/HLA-DR^+^/CD64^+^/MerTK^+^ MΦ from tumor and healthy tissue. 

Cells were harvested for AAS (5000 cells) or used for RNA isolation (1000 cells). RNA isolation and transcription were performed using the RNeasy Micro Kit (Qiagen, 74004) and Sensiscript RT Kit (Qiagen, 205211) according to the manufacturer’s kit protocols. 

### 4.9. Cell Culture

Human renal cancer cell lines CAKI-1 and 786-O cells (kindly provided by PD Dr. Anja Urbschat) were cultured in Dulbecco’s modified Eagle’s medium (Gibco, Dreieich, Germany, 41965) supplemented with penicillin 100 U/mL (Sigma-Aldrich, P4333), streptomycin 100 mg/mL (Sigma-Aldrich, S8636), and 10% FCS (Capricorn Scientific, Ebersdorfergrund, Germany FBS-11A). Cells were regularly tested for mycoplasma contamination using Venor GeM Classic (Minerva Biolabs, Berlin, Germany, 11-1100).

### 4.10. Tumor Tubular Epithelial Cell Isolation

Human tubular epithelial cells (TTEC) were isolated as previously described [52]. Briefly, tumor tissue was minced, digested with collagenase/dispase (1 mg/mL), and passed through a 106 µm mesh. The tumor tissue solution was then incubated with collagenase (1 mg/mL), DNase (0.1 mg/mL) and MgCl2 (5 mmol/L). Cells were seeded on FCS-precoated plates and grown in M199 medium (Sigma Aldrich, M4530), supplemented with penicillin (100 U/mL), streptomycin (100 mg/mL), and 10% FBS. Meropenem (100 µg/mL, Sigma Aldrich, M2574) was added to the culture medium for the first 2–3 days after isolation. Passages from two to four were used for experiments.

### 4.11. EC Fluids Generation

Frozen tumor and adjacent healthy renal tissues were crushed into fragments <2 mm in diameter and suspended in 1:2 weight/volume of 2× phosphate-buffered saline (PBS). The solution was rotated at 4 °C for 3 h. The samples were then vortexed, and the centrifugation-cleared supernatants were used for experiments.

### 4.12. Generation of Conditioned Medium (CM) from Human MΦ

Human monocytes were isolated from commercially available, anonymized buffy coats (DRK-Blutspendedienst Baden-Württemberg-Hessen, Frankfurt, Germany) using Ficoll-Hypaque gradients (PAA Laboratories, Cölbe, Germany) as previously described [41]. Briefly, monocytes were differentiated into primary human MΦ with RPMI-1640 containing 5% AB-positive human serum (DRK-Blutspendedienst Baden-Württemberg-Hessen, Frankfurt, Germany). Prior to stimulation, cells were serum-starved for 24 h and stimulated with 20 ng/mL IL-10 (Peprotech, Hamburg, Germany) for 24 h to generate conditioned-media of polarized MΦ [41]. Conditioned-media from iron-releasing MΦ were collected and used for following proliferation and migration assays. Supernatant of unstimulated MΦ served as control.

### 4.13. Proliferation and Migration Assays

Proliferation and migration assays were performed using the xCELLigence RTCA DP instrument (OLS, Bremen, Germany) as previously described [53]. Proliferation was recorded continuously for 3 days and migration for 24 h. Data were acquired as a measure for time-dependent impedance changes. RTCA Software 1.2 (OLS) was used for acquisition and analysis.

### 4.14. Immunohistochemistry

Immunohistochemical staining was adapted from previously described protocols [35]. Formalin-fixed and paraffin-embedded patient tissues were stained with antibodies against FPN (Novus, NBP1-21502) and CD163 (Abcam, Cambridge, UK, ab182422) according to the manufacturer’s protocol using the Opal 4-color-automation IHC-kit (PerkinElmer, Rodgau, Germany, NEL820001KT). Images were acquired using the LSM 800 microscope (Zeiss) and edited using ImageJ software. 

### 4.15. Hematoxylin and Eosin Stain

For hematoxylin and eosin staining, formalin-fixed and paraffin-embedded tissues were rehydrated, stained using Mayer’s hemalum solution (Merck, 109249), washed, counter-stained using Eosin (Merck, 102439), and mounted in Entellan (Merck, 107961). An Axioskop 40 (Zeiss) was used to acquire images.

### 4.16. XTT

Cytotoxicity of iron chelators was tested by a photometric XTT assay (Panreac, Darmstadt, Germany, A8088). Briefly, sub-confluent cells were exposed to iron chelators for 12 h. Subsequently, XTT reagent was added and absorbance was measured at 450 nm vs. 630 nm according to manufacturer’s protocol. Experiments were conducted in quintuplicates. Cell viability was normalized to the untreated control.

### 4.17. Synthesis and Chemical Characterization of the Extracellular Chelator EC1

2-Hydroxybenzaldehyde (125 mg, 1.0 mmol) was added to a solution of sodium 4-(hydrazinecarbothioamido) benzenesulfonate (426 mg, 1.5 mmol) in water (1 mL). Ethanol (2 mL) was added and the solution was brought to reflux and stirred for 30 min. The reaction mixture was then allowed to cool to room temperature and the formed precipitate was filtered, washed with ethanol, and dried under vacuum. The identity and purity of the desired product (311 mg, 81% yield) were confirmed by high-resolution mass spectrometry via electrospray ionization (HRMS-ESI) and nuclear magnetic resonance (NMR) spectroscopy. HRMS-ESI (*m/z*): [M – Na]^−^ calcd for [C_14_H_12-_N_3_O_4_S_2_]^−^, 350.02747; found, 350.02741; [M + H]^+^ calcd for [C_14_H_13-_N_3_NaO_4_S_2_]^+^, 374.02397; found, 374.02401. ^1^H NMR (500 MHz, DMSO-*d*_6_) δ 11.79 (s, 1H), 10.07 (s, 1H), 9.98 (bs, 1H), 8.50 (s, 1H), 8.09 (bd, 1H), 7.64–7.44 (m, 4H), 7.24 (m, 1H), 6.95–6.78 (m, 2H). ^13^C NMR (125 MHz, DMSO-*d*_6_) δ 176.21, 157.17, 145.37, 140.65, 139.76, 131.86, 127.55, 125.85, 125.12, 120.69, 119.70, 116.53.

### 4.18. Chelator Solutions

2,2’-Dipyridine (2’2-DPD) was obtained commercially (Sigma Aldrich, D216305) and the intracellular prochelator (TC3-S)_2_ was prepared as previously reported [54]. Extracellular chelator EC1 was synthesized as described above. For experiments in cell cultures, stock solutions were prepared at a standard concentration of 100 µM in dimethyl sulfoxide (DMSO) and were always prepared freshly in degassed DMSO.

### 4.19. Statistical Analysis

Statistical analysis was performed applying GraphPad Prism software (GraphPad Inc., San Diego, CA, USA, version 8.2.1). Variable distribution was tested for normality using the Kolmogorov-Smirnov test. Respectively, Gaussian distributed, and non-Gaussian distributed patient samples were statistical analyzed using two-tailed paired student’s *t*-test or Wilcoxon matched-pairs signed-ranks test. In vitro experiments were analyzed using one-way ANOVA. Cell culture experiments were performed at least three times (independent experiments using technical replicates). Patient samples were used in experiments upon availability. P values were considered significant at * *p* < 0.05, ** *p* < 0.01, *** *p* < 0.001. 

## 5. Conclusions

This study provides new insights of a significantly altered iron metabolism in renal cell carcinoma. Most of the studied iron-regulated genes are associated with tumor grade and tumor pT-stage. Moreover, our results suggest that tumor-associated macrophages adopt a pro-tumorigenic iron-releasing phenotype through increased expression of FPN. These tumor-associated macrophages are then able to fuel the increased iron demands of tumor cells by secreting iron in the tumor microenvironment. 

EC1, a novel iron chelator, specifically scavenges iron in the extracellular space and was able to reverse pro-tumorigenic effects of macrophage-conditioned media on proliferation and migration of cancer cells, including primary patient-derived renal cancer cells. These results might pave the way towards further in vivo studies addressing the possibility to interfere with iron availability in the tumor microenvironment by targeted chelation strategies. 

## 6. Patents

Issued patent: Tomat E.; Chang, T. M. “Redox-Directed Chelators Targeting Intracellular Metal Ions” U.S. Patent No. 9,486,423, November 8th, 2016.

Pending patent application: Tomat, E.; Chang, T.; Akam, E. A. “Redox-activated Pro-chelators” U.S. Patent Appl. No.: 16/200,286, November 26th, 2018.

## Figures and Tables

**Table 1:** Patient cohort. The patient cohort is composed of 64 patients, grouped into three major renal tumor types ccRCC, pRCC, and chRCC. Patient parameters age, sex, pT-stage and grade are depicted in the table.

Number of Patients	ccRCC	pRCC	chRCC
	56	7	7
**Age (years)**			
mean	64 ± 10	68 ± 11	63 ± 10
median	64 ± 10	71 ± 11	62 ± 10
range	44–85	48–79	48–75
**sex**			
female	24%	29%	80%
male	76%	71%	20%
**pT-stage**			
pT1-pT2	55%		
pT3-pT4	45%		
**Grade**			
G1-G2	84%		
G3-G4	16%		

## Supplementary Materials

The following are available online at https://www.mdpi.com/2072-6694/12/3/530/s1, Figure S1: Primary human renal tissue verification, Figure S2: Perl’s staining of histopathological renal cell carcinoma subtypes. Figure S3: Characterization of the extracellular iron chelator EC1.

## References

[1] White M.F., Dillingham M.S. (2012). Iron-sulphur clusters in nucleic acid processing enzymes. Curr. Opin. Struct. Biol..

[2] Wang Y., Yu L., Ding J., Chen Y. (2018). Iron Metabolism in Cancer. Int. J. Mol. Sci..

[3] Pantopoulos K., Porwal S.K., Tartakoff A., Devireddy L. (2012). Mechanisms of mammalian iron homeostasis. Biochemistry.

[4] Haase V.H. (2010). Hypoxic regulation of erythropoiesis and iron metabolism. Am. J. Physiol. Ren. Physiol..

[5] Seminog O.O., Ogunlaja O.I., Yeates D., Goldacre M.J. (2016). Risk of individual malignant neoplasms in patients with sickle cell disease: English national record linkage study. J. R. Soc. Med..

[6] Vargas-Olvera C.Y., Sánchez-González D.J., Solano J.D., Aguilar-Alonso F.A., Montalvo-Muñoz F., Martínez-Martínez C.M., Medina-Campos O.N., Ibarra-Rubio M.E. (2012). Characterization of N-diethylnitrosamine-initiated and ferric nitrilotriacetate-promoted renal cell carcinoma experimental model and effect of a tamarind seed extract against acute nephrotoxicity and carcinogenesis. Mol. Cell. Biochem..

[7] Greene C.J., Attwood K., Sharma N.J., Gross K.W., Smith G.J., Xu B., Kauffman E.C. (2017). Transferrin receptor 1 upregulation in primary tumor and downregulation in benign kidney is associated with progression and mortality in renal cell carcinoma patients. Oncotarget.

[8] Bray F., Ferlay J., Soerjomataram I., Siegel R.L., Torre L.A., Jemal A. (2018). Global cancer statistics 2018: GLOBOCAN estimates of incidence and mortality worldwide for 36 cancers in 185 countries. CA Cancer J. Clin..

[9] Makhov P., Joshi S., Ghatalia P., Kutikov A., Uzzo R.G., Kolenko V.M. (2018). Resistance to Systemic Therapies in Clear Cell Renal Cell Carcinoma: Mechanisms and Management Strategies. Mol. Cancer Ther..

[10] Gill D.M., Agarwal N., Vaishampayan U. (2017). Evolving Treatment Paradigm in Metastatic Renal Cell Carcinoma. Am. Soc. Clin. Oncol. Educ. Book.

[11] Amin A., Hammers H. (2018). The Evolving Landscape of Immunotherapy-Based Combinations for Frontline Treatment of Advanced Renal Cell Carcinoma. Front. Immunol..

[12] Cella D., Grünwald V., Escudier B., Hammers H.J., George S., Nathan P., Grimm M.-O., Rini B.I., Doan J., Ivanescu C. (2019). Patient-reported outcomes of patients with advanced renal cell carcinoma treated with nivolumab plus ipilimumab versus sunitinib (CheckMate 214): A randomised, phase 3 trial. Lancet Oncol..

[13] Kohgo Y., Ikuta K., Ohtake T., Torimoto Y., Kato J. (2008). Body iron metabolism and pathophysiology of iron overload. Int. J. Hematol..

[14] Torti S.V., Torti F.M. (2013). Iron and cancer: More ore to be mined. Nat. Rev. Cancer.

[15] Kirkali Z., Esen A.A., Kirkali G., Güner G. (1995). Ferritin: A tumor marker expressed by renal cell carcinoma. Eur. Urol..

[16] Jézéquel P., Campion L., Spyratos F., Loussouarn D., Campone M., Guérin-Charbonnel C., Joalland M.-P., André J., Descotes F., Grenot C. (2012). Validation of tumor-associated macrophage ferritin light chain as a prognostic biomarker in node-negative breast cancer tumors: A multicentric 2004 national PHRC study. Int. J. Cancer.

[17] Brookes M.J., Hughes S., Turner F.E., Reynolds G., Sharma N., Ismail T., Berx G., McKie A.T., Hotchin N., Anderson G.J. (2006). Modulation of iron transport proteins in human colorectal carcinogenesis. Gut.

[18] Wang W., Deng Z., Hatcher H., Miller L.D., Di X., Tesfay L., Sui G., D’Agostino R.B., Torti F.M., Torti S.V. (2014). IRP2 regulates breast tumor growth. Cancer Res..

[19] Ludwig H., Müldür E., Endler G., Hübl W. (2013). Prevalence of iron deficiency across different tumors and its association with poor performance status, disease status and anemia. Ann. Oncol..

[20] Corna G., Campana L., Pignatti E., Castiglioni A., Tagliafico E., Bosurgi L., Campanella A., Brunelli S., Manfredi A.A., Apostoli P. (2010). Polarization dictates iron handling by inflammatory and alternatively activated macrophages. Haematologica.

[21] Cairo G., Recalcati S., Mantovani A., Locati M. (2011). Iron trafficking and metabolism in macrophages: Contribution to the polarized phenotype. Trends Immunol..

[22] Recalcati S., Locati M., Marini A., Santambrogio P., Zaninotto F., De Pizzol M., Zammataro L., Girelli D., Cairo G. (2010). Differential regulation of iron homeostasis during human macrophage polarized activation. Eur. J. Immunol..

[23] Murray P.J., Wynn T.A. (2011). Protective and pathogenic functions of macrophage subsets. Nat. Rev. Immunol..

[24] Chevrier S., Levine J.H., Zanotelli V.R.T., Silina K., Schulz D., Bacac M., Ries C.H., Ailles L., Jewett M.A.S., Moch H. (2017). An Immune Atlas of Clear Cell Renal Cell Carcinoma. Cell.

[25] Chanmee T., Ontong P., Konno K., Itano N. (2014). Tumor-associated macrophages as major players in the tumor microenvironment. Cancers.

[26] Ikemoto S., Yoshida N., Narita K., Wada S., Kishimoto T., Sugimura K., Nakatani T. (2003). Role of tumor-associated macrophages in renal cell carcinoma. Oncol. Rep..

[27] Toge H., Inagaki T., Kojimoto Y., Shinka T., Hara I. (2009). Angiogenesis in renal cell carcinoma: The role of tumor-associated macrophages. Int. J. Urol..

[28] Lewis C.E., Pollard J.W. (2006). Distinct role of macrophages in different tumor microenvironments. Cancer Res..

[29] Komohara Y., Hasita H., Ohnishi K., Fujiwara Y., Suzu S., Eto M., Takeya M. (2011). Macrophage infiltration and its prognostic relevance in clear cell renal cell carcinoma. Cancer Sci..

[30] Xu L., Zhu Y., Chen L., An H., Zhang W., Wang G., Lin Z., Xu J. (2014). Prognostic value of diametrically polarized tumor-associated macrophages in renal cell carcinoma. Ann. Surg. Oncol..

[31] Sangaletti S., Di Carlo E., Gariboldi S., Miotti S., Cappetti B., Parenza M., Rumio C., Brekken R.A., Chiodoni C., Colombo M.P. (2008). Macrophage-derived SPARC bridges tumor cell-extracellular matrix interactions toward metastasis. Cancer Res..

[32] Sandlund J., Oosterwijk E., Grankvist K., Oosterwijk-Wakka J., Ljungberg B., Rasmuson T. (2007). Prognostic impact of carbonic anhydrase IX expression in human renal cell carcinoma. BJU Int..

[33] Nguyen D.P., Vertosick E.A., Corradi R.B., Vilaseca A., Benfante N.E., Touijer K.A., Sjoberg D.D., Russo P. (2016). Histological subtype of renal cell carcinoma significantly affects survival in the era of partial nephrectomy. Urol. Oncol..

[34] Marques O., Porto G., Rêma A., Faria F., Cruz Paula A., Gomez-Lazaro M., Silva P., Martins da Silva B., Lopes C. (2016). Local iron homeostasis in the breast ductal carcinoma microenvironment. BMC Cancer.

[35] Mertens C., Mora J., Ören B., Grein S., Winslow S., Scholich K., Weigert A., Malmström P., Forsare C., Fernö M. (2018). Macrophage-derived lipocalin-2 transports iron in the tumor microenvironment. Oncoimmunology.

[36] Jung M., Weigert A., Tausendschön M., Mora J., Ören B., Sola A., Hotter G., Muta T., Brüne B. (2012). Interleukin-10-induced neutrophil gelatinase-associated lipocalin production in macrophages with consequences for tumor growth. Mol. Cell. Biol..

[37] Mertens C., Akam E.A., Rehwald C., Brüne B., Tomat E., Jung M. (2016). Intracellular Iron Chelation Modulates the Macrophage Iron Phenotype with Consequences on Tumor Progression. PLoS ONE.

[38] Yu Y., Kalinowski D.S., Kovacevic Z., Siafakas A.R., Jansson P.J., Stefani C., Lovejoy D.B., Sharpe P.C., Bernhardt P.V., Des Richardson R. (2009). Thiosemicarbazones from the old to new: Iron chelators that are more than just ribonucleotide reductase inhibitors. J. Med. Chem..

[39] Utterback R.D., Tomat E. (2019). Developing Ligands to Target Transition Metals in Cancer. Encycl. Inorg. Bioinorg. Chem..

[40] Akam E.A., Chang T.M., Astashkin A.V., Tomat E. (2014). Intracellular reduction/activation of a disulfide switch in thiosemicarbazone iron chelators. Metallomics.

[41] Akam E.A., Utterback R.D., Marcero J.R., Dailey H.A., Tomat E. (2018). Disulfide-masked iron prochelators: Effects on cell death, proliferation, and hemoglobin production. J. Inorg. Biochem..

[42] Greene B.T., Thorburn J., Willingham M.C., Thorburn A., Planalp R.P., Brechbiel M.W., Jennings-Gee J., Wilkinson J., Torti F.M., Torti S.V. (2002). Activation of caspase pathways during iron chelator-mediated apoptosis. J. Biol. Chem..

[43] Jung M., Mertens C., Tomat E., Brüne B. (2019). Iron as a Central Player and Promising Target in Cancer Progression. Int. J. Mol. Sci..

[44] Alkhateeb A.A., Han B., Connor J.R. (2013). Ferritin stimulates breast cancer cells through an iron-independent mechanism and is localized within tumor-associated macrophages. Breast Cancer Res. Treat..

[45] Kukulj S., Jaganjac M., Boranic M., Krizanac S., Santic Z., Poljak-Blazi M. (2010). Altered iron metabolism, inflammation, transferrin receptors, and ferritin expression in non-small-cell lung cancer. Med. Oncol..

[46] Nseyo U.O., Williams P.D., Murphy G.E. (1986). Clinical significance of erythropoietin levels in renal carcinoma. Urology.

[47] Dowd A.A., Ibrahim F.I., Mohammed M.M. (2014). Renal cell carcinoma as a cause of iron deficiency anemia. Afr. J. Urol..

[48] Jiang X.P., Elliott R.L., Head J.F. (2010). Manipulation of iron transporter genes results in the suppression of human and mouse mammary adenocarcinomas. Anticancer Res..

[49] Coombs G.S., Schmitt A.A., Canning C.A., Alok A., Low I.C.C., Banerjee N., Kaur S., Utomo V., Jones C.M., Pervaiz S. (2012). Modulation of Wnt/β-catenin signaling and proliferation by a ferrous iron chelator with therapeutic efficacy in genetically engineered mouse models of cancer. Oncogene.

[50] Hrabinski D., Hertz J.L., Tantillo C., Berger V., Sherman A.R. (1995). Iron repletion attenuates the protective effects of iron deficiency in DMBA-induced mammary tumors in rats. Nutr. Cancer.

[51] Brierley J., Gospodarowicz M.K., Wittekind C., Brierley J., Gospodarowicz M.K., Wittekind C., O’Sullivan B. (2017). TNM Classification of Malignant Tumours.

[52] Baer P.C., Nockher W.A., Haase W., Scherberich J.E. (1997). Isolation of proximal and distal tubule cells from human kidney by immunomagnetic separation. Technical note. Kidney Int..

[53] Jung M., Ören B., Mora J., Mertens C., Dziumbla S., Popp R., Weigert A., Grossmann N., Fleming I., Brüne B. (2016). Lipocalin 2 from macrophages stimulated by tumor cell-derived sphingosine 1-phosphate promotes lymphangiogenesis and tumor metastasis. Sci. Signal..

[54] Chang T.M., Tomat E. (2013). Disulfide/thiol switches in thiosemicarbazone ligands for redox-directed iron chelation. Dalton Trans..

